# A Poisson regression approach for assessing morbidity risk and determinants among under five children in Nigeria

**DOI:** 10.1038/s41598-024-72373-4

**Published:** 2024-09-16

**Authors:** Idika E. Okorie, Emmanuel Afuecheta, Saralees Nadarajah, Adaoma Bright, Anthony C. Akpanta

**Affiliations:** 1 Department of Mathematics, Khalifa University, P. O. Box 127788, Abu Dhabi, UAE; 2 Department of Mathematics and Statistics, King Fahd University of Petroleum and Minerals, Dhahran, Saudi Arabia; 3 Department of Mathematics, University of Manchester, Manchester, M13 9PL UK; 4 Birmingham City Council, 10 Woodcock Street, Birmingham, B7 4BL UK; 5 Department of Statistics, Abia State University, Uturu, Abia State Nigeria

**Keywords:** Morbidity, Nigeria, Poisson regression, Risk analysis, Medical research, Risk factors, Mathematics and computing

## Abstract

In this paper, we have provided more insights on the relationship between under five morbidity in Nigeria and some background characteristics using a Poisson regression model and the most recent 2018 NDHS data on Acute Respiratory Infection (ARI), diarrhoea and fever. Some of our results are that children 36–47 months old have the highest risk of ARI [OR = 1.45; CI (1.31,1.60)] while children less than 6 months old have the lowest risk of ARI [OR = 0.14; CI (0.11,0.17)]. The prevalence of diarrhoea is generally high among children under 48-59 months old but highest among children 6–11 months old [OR = 4.34; CI (3.69,5.09)]. Compared to children 48–59 months old, children in all other age categories except 24–34 months old have a high risk of fever [OR = 0.95; CI (0.73,1.24)]. ARI is more prevalent among female children [OR = 8.88; CI (8.02,9.82)] while diarrhoea [OR = 21.75; (19.10,24.76)] and fever [OR = 4.78; CI (4.31,5.32)] are more prevalent among male children. Children in urban areas are more likely to suffer ARI [OR = 9.49; CI (8.31,10.85)] while children in rural areas are more likely to suffer both diarrhoea [OR = 21.75; CI (19.10,24.76)] and fever [OR = 4.90; CI (4.26,5.63)]. Children in the South-South have the highest risk of ARI [OR = 4.03; CI (3.65,4.454)] while children in the North Central have the lowest risk of ARI [OR = 1.55; CI (1.38,1.74)] and highest risk of diarrhoea [OR = 3.34; CI (2.30,5.11)]. Children in the Northeast have the highest risk of fever [OR = 1.30; CI (1.14,1.48)]. In the Northcentral region, Kogi state has the highest prevalence of fever [OR = 2.27; CI (1.62,3.17)], while Benue state has the lowest [OR = 0.35; CI (0.20,0.60)]. Children in Abuja state face similar risks of fever and diarrhoea [OR = 0.84; CI (0.55,1.27)], with the risk of diarrhoea in Abuja being comparable to that in Plateau state [OR = 1.57; CI (0.92,2.70)]. Nasarawa state records the highest incidence of diarrhoea in the Northcentral [OR = 5.12; CI (3.03,8.65)], whereas Kogi state reports the lowest [OR = 0.29; CI (0.16,0.53)]. In the Northeast, Borno state has the highest rate of fever [OR = 3.28; CI (2.80,3.84)], and Bauchi state the lowest [OR = 0.38; CI (0.29,0.50)]. In Adamawa state, the risks of fever and diarrhoea are nearly equivalent [OR = 1.17; CI (0.97,1.41)], and the risk of fever there is similar to that in Taraba state [OR = 0.92; CI (0.75,1.12)]. Diarrhoea is most prevalent in Yobe state [OR = 3.17; CI (2.37,4.23)] and least prevalent in Borno state [OR = 0.26; CI (0.20,0.33)]. In the Northwest, the risk of fever is similarly high in Zamfara and Kebbi states [OR = 1.04; CI (0.93,1.17)], with Kastina state showing the lowest risk [OR = 0.39; CI (0.34,0.46)]. Children in Zamfara state experience notably different risks of fever and diarrhoea [OR = 0.07; CI (0.05,0.10)]. Kaduna state reports the highest incidence of diarrhoea [OR = 21.88; CI (15.54,30.82)], while Kano state has the lowest [OR = 2.50; CI (1.73,3.63)]. In the Southeast, Imo state leads in fever incidence [OR = 8.20; CI (5.61,11.98)], while Anambra state has the lowest [OR = 0.40; CI (0.21,0.78)]. In Abia state, the risk of fever is comparable to that in Enugu state [OR = 1.03; CI (0.63,1.71)], but the risks of fever and diarrhoea in Abia differ significantly [OR = 2.67; CI (1.75,4.06)]. Abia state also has the highest diarrhoea rate in the Southeast [OR = 2.67; CI (1.75,4.06)], with Ebonyi state having the lowest [OR = 0.05; CI (0.03,0.09)]. In the South-South region, Bayelsa and Edo states have similar risks of fever [OR = 1.28; CI (0.84,1.95)], with Akwa Ibom state reporting the highest fever rate [OR = 4.62; CI (3.27,6.52)] and Delta state the lowest [OR = 0.08; CI (0.02,0.25)]. Children in Bayelsa state face distinctly different risks of fever and diarrhoea [OR = 0.56; CI (0.34,0.95)]. Rivers state shows the highest incidence of diarrhoea in the South-South [OR = 10.50; CI (4.78,23.06)], while Akwa Ibom state has the lowest [OR = 0.30; CI (0.15,0.57)]. In the Southwest, Lagos and Osun states have similar risks of fever [OR = 1.00; CI (0.59,1.69)], with Ogun state experiencing the highest incidence [OR = 3.47; CI (2.28,5.28)] and Oyo state the lowest [OR = 0.18; CI (0.07,0.46)]. In Lagos state, the risks of fever and diarrhoea are comparable [OR = 0.96; CI (0.57,1.64)], and the risk of diarrhoea is similar to those in Ekiti, Ogun, and Ondo states. Oyo state has the highest diarrhoea rate in the Southwest [OR = 10.99; CI (3.81,31.67)], with Ogun state reporting the lowest [OR = 0.77; CI (0.42,1.42)]. Children of mothers with more than secondary education are significantly less likely to suffer ARI [OR = 0.35; CI (0.29,0.42)], whereas children of mothers without any education run a higher risk of diarrhoea [OR = 2.12; CI (1.89,2.38)] and fever [OR = 2.61; CI (2.34,2.91)]. Our analysis also indicated that household wealth quintile is a significant determinant of morbidity. The results in this paper could help the government and non-governmental agencies to focus and target intervention programs for ARI, diarrhoea and fever on the most vulnerable and risky under five groups and populations in Nigeria.

## Introduction

It is on record that the mortality rate for children under the age of five has significantly plummeted globally by more than half in the last three decades. This can be likened to 1 in 11 children dying before reaching age five in 1990 as against 1 in 27 in 2019^1,2^.

Sub-Saharan Africa is one of the regions where the global under five deaths are still high, with more than 2.5 million deaths annually, reaching up to 50% of the 5.3 million under five deaths in 2019 and accounting for nearly three-fifths of all the countries grappling with high under five mortality^3,4^. Although some countries in this region, such as Rwanda and Botswana, have made a major leap forward and have saved souls, many other countries within the region are still struggling^5^. The most recent report indicates that Nigeria, the Democratic Republic of the Congo, Ethiopia, the Republic of Tanzania, and Angola are among the top 10 countries with the highest number of deaths for children under 5 years old^3^. Out of these five countries, Nigeria tops the chart with over 800 thousand under five deaths in 2019.

Significant efforts have been made to slow down global infant mortality. The progress made so far could be attributed to the unfaltering application of resources and knowledge to public health as well as global health initiatives such as the goal-3 (i.e., health and well-being agenda) of the Sustainable Development Goals (SDGs) of the United Nations (UNICEF, 2015). Given this headway, under five mortality worldwide was estimated to be around 5 million in 2020, a marked reduction from the 2019 figure. Out of this estimate, more than half (above 2.9 million) of the number occurred in the developing countries, where Nigeria still topped the chart by accounting for about 844,000 of the under five deaths, and the vast majority of these deaths were from avoidable and curable causes^3,6^.

The high under five mortality in Nigeria is due to diseases such as malaria, diarrhoea, and respiratory tract infections^7–9^. According to Odejimi et al.^10^, the three major causes of under five mortality in Nigeria are malaria (35%), diarrhoea (23%), and pneumonia (12%). These diseases account for most outpatient children consultations as well as children’s hospital admissions. According to Adedokun and Yaya^3^, malaria and diarrhoea account for 50% of all admissions into children’s hospitals and wards, while respiratory and related infections account for 19% of all admissions into children’s hospitals and wards in Nigeria.

Different schemes and programs involving millions of dollars have been initiated to combat these diseases. For instance, the Global Technical Strategy for Malaria was inaugurated in 2015 by the World Health Organization (WHO) with the aim of reducing malaria to the barest minimum by 2030 across the globe. As one of the countries with the greatest malaria burden, Nigeria adopted the scheme. However, despite this and other related efforts, childhood morbidity remains a great burden and a major public health concern in the country. Furthermore, several studies have identified variables or factors that sway under five morbidity. Some of these include, but are not limited to, child gender, residence, maternal education, and household wealth. Others include the level of hygiene practice, cultural affiliation (region), and ethnic differentials. Adedini et al.^11^ observed significant differentials in under five mortality by ethnic regions or affiliations.

Nigeria has 36 states and six geopolitical zones. It is the most populous African country, with over 200 million people. Ineffective and unpatriotic leadership are among the major problems in the country, which has led to poor growth and development across all sectors of the economy, including health care, with the ripple effect hitting hard on the ordinary citizen. That said, the country is seriously struggling to eliminate the causes of under five mortality, such as diarrhoea, malaria, fever, and respiratory infection. Given that under five mortality remains unseemly high in Nigeria despite all the concerted efforts and the adoption of several recommended schemes by different international bodies such as WHO, it is important to study the pattern of morbidity and mortality of under five children based on specific background characteristics. This paper focuses on assessing the relationship between under five morbidity and some background characteristics in Nigeria while accounting for state or regional differentials. To the best of our knowledge, we are not aware of any paper that has utilized a Poisson regression model to study under five morbidity in Nigeria. The major contributions of this paper are: (i) to provide a statistical analysis of factors that influence morbidity and compare risk exposure among children aged 5 years and under in Nigeria; and (ii) to quantify and compare the risk of diarrhoea and fever among under five children between different states in all six geopolitical zones in Nigeria.

The contents of this paper are organized as follows. “Data” section describes the data used in our analysis. In “Methods” section, we briefly give the methodology and models used. “Analysis and discussion of results” section outlines our results and provides a discussion of them. “Conclusions” section 5 provides a conclusion and summary of our results. The limitations of the paper are highlighted in “Limitations” section.

## Data

Data were extracted from the 2018 Nigeria Demographic and Health Survey (2018 NDHS) report (National Population Commission - NPC/Nigeria and ICF,^12^), see https://www.dhsprogram.com/pubs/pdf/SR264/SR264.pdf. The publication is freely available in the public domain. The 2018 NDHS report contains the most representative and up-to-date data on Nigeria’s demographic and health indicators. The first-hand data was collected by the National Population Commission (NPC) Nigeria by questionnaire method across Nigeria from 14 August to 29 December 2018. The requirement for ethical compliance was met and the approval was explicitly declared in the report “*The survey protocol was reviewed and approved by the National Health Research Ethics Committee of Nigeria (NHREC) and the ICF Institutional Review Board.*”

The 2018 NDHS data is based on a sample of 42,000 households, and it uses a two-stage stratified cluster sampling technique. The data in this paper pertains to mothers aged between 15 and 49 and their children aged between 0 and 59 months (under 5 years old) in the sample households. More information on the questionnaire, the sampling design, and techniques, and the data can be found in the 2018 NDHS report. In this paper, we have only used the data on the weighted number of children under the age of five who had symptoms of acute respiratory infection (ARI), fever, and diarrhoea in the 2 weeks preceding the survey according to some relevant background characteristics like age (in months), gender, residence (urban and rural), geopolitical zone, mothers education level, state and wealth quintile.

The weighted data was necessary because the Demographic and Health Surveys (DHS) usually involve complicated sampling techniques capable of rendering standard statistical methods inadequate for analyzing data from such complex sampling schemes due to high cluster effects primarily induced by the sampling design.

The ARIs represent all the infections of the lower and upper respiratory tracts^13^. The ARIs are a major cause of morbidity and mortality worldwide^14^. According to Denny^15^, the most common upper respiratory diseases are ear infections, rhinitis, sinusitis, laryngitis, acute pharyngitis or tonsillopharyngitis, laryngitis, and epiglottitis. Viruses are responsible for a large number of upper respiratory infections, for instance, rhinoviruses are responsible for about 25–30% of upper respiratory infections, while the respiratory syncytial viruses such as the parainfluenza and influenza viruses, human metapneumovirus and adenoviruses account for 25–35% of upper respiratory infections; the coronaviruses account for 10% of the upper respiratory infections, and the rest of the upper respiratory infections are due to unknown viruses. The most common lower respiratory infections in children are pneumonia and bronchiolitis which are caused by respiratory syncytial viruses.

Fever is a response to infection, disease, inflammation, and trauma^16^. Fever is a common symptom of malaria^17,18^. According to Penn Medicine, see https://www.pennmedicine.org/for-patients-and-visitors/patient-information/conditions-treated-a-to-z/fever, fever is defined as the temporary rise in the body’s temperature due to disease or illness. Specifically, children have a fever when their rectal temperature is 100.4F (38C) or above, the oral temperature is 99.5F (37.5C) or above and the axillary temperature is 99F (37.2C) or above. Several factors, like the type of thermometer and the body site where measurement is taken, could influence temperature readings in humans. Therefore, there is no generally acceptable temperature benchmark for fever definition^19^.

Recently, diarrhoea has been defined as more than three liquid bowel movements per day and the Bristol Stool Chart score of 7^20^. Other definitions of diarrhoea can be found in Gidudu et al.^21^. Diarrhoea alters the microbiome and, consequently, the immune system. It is a significant cause of morbidity and mortality in young children in Nigeria^22^. According to UNICEF, see https://data.unicef.org/topic/child-health/diarrhoeal-disease/, despite the wide availability of diarrhoea treatments, it still remains one of the leading causes of infant mortality. Diarrhoea was responsible for about 9% of global under five deaths in 2021, suggesting that more than 1200 children die daily, or approximately 444,000 children die annually from diarrhoea. The under five deaths due to diarrhoea are highest in South Asia and sub-Saharan Africa. diarrhoea can simply be treated by oral rehydration therapy (ORT) such as oral rehydration salts (ORS) and some recommended homemade fluids. Just like fever, diarrhoea could manifest as a symptom of other life-threatening diseases such as COVID-19^23^.

In this paper, there are 814 (6.66%) children who had experienced ARI, 7466 (61.05%) children who had experienced fever, and 3950 (32.30%) children who had experienced diarrhoea.

## Methods

Suppose we classify *n* children less than 5 years old according to some background characteristics (*I*) and the type of morbidity (*J*) they have experienced. Let $x_{i, j}$ be the number of children that fall into the *i*th category of certain background characteristics and *j*th category of certain morbidity. The data $\left\{ x_{i, j}\right\}$ form a two-dimensional contingency table. We can fit a Poisson model to the contingency table data and test the association between the row and column classifications. The Poisson regression model can be specified as

1 $$\begin{aligned} \displaystyle x_{i, j}=\mu _{i, j}+\xi _{i, j}\sim \textrm{Poisson}\left( \mu _{i, j}\right) \ \textrm{independent}, \end{aligned}$$

where

2 $$\begin{aligned} \displaystyle \log \left( \mu _{i, j}\right) =\log \left( n\theta _{i, j}\right) =c+\alpha _i+\beta _j+\lambda _{i, j}, \ \displaystyle i=1,\ldots ,I, \ \displaystyle j=1,\ldots ,J, \end{aligned}$$

where *c* denotes the intercept, $\alpha _i$ denotes the coefficient of *i*th row category, $\beta _j$ denotes the coefficient of the *j*th column category, $\lambda _{i,j}$ denotes the coefficient of the interaction between the *i*th row category and the *j*th column category, $\theta _{i,j}$ denotes the cell probability (i.e., the probability that a randomly selected subject belongs to the *i*th row and *j*th column) and $\xi _{i, j}$ denotes the error term.

## Analysis and discussion of results

We fitted the Poisson regression model in (1) to different combinations of categories involving morbidity and each one of the background characteristics: children’s age, gender, residence, geopolitical zone, mothers’ education level, state, and wealth quintile. For the Poisson regression, there are two possible models, namely the additive model (without the interaction term in (1)), also known as the null model, and the multiplicative model ((1)) also known as the full model. The additive model is equivalent to the chi-square test for independence; it is useful for investigating whether an association exists between the categories. The multiplicative model can tell us more about the relationships. In each case, the two models were fitted. However, for simplicity, we only report the complete results for the multiplicative model. In all the scenarios, the additive model was rejected based on the deviance analysis in favor of the multiplicative model, as we report next.

We chose and fitted the multiplicative models in Table 1 because the additive model for children’s age and morbidity with a deviance of 562.96 on 10 degrees of freedom (df) is significantly different from the full model ($\chi ^2_{0.05;10}=18.31$); hence, rejecting the null model of no association between children age and morbidity. The additive model for children’s gender and morbidity with a deviance of 4756.6 on 2 df is significantly different from the full model ($\chi ^2_{0.05;2}=5.99$); thus, rejecting the null model of no association between children’s gender and morbidity. The additive model for residence and morbidity with a deviance of 2771.7 on 2 df is significantly different from the full model ($\chi ^2_{0.05;2}=5.99$); thus, rejecting the null model of no association between residence and morbidity. The additive model for zone and morbidity with a deviance of 4255.6 on 10 df is significantly different from the full model ($\chi ^2_{0.05;10}=18.31$); thus, rejecting the null model of no association between geopolitical zone and morbidity. The additive model for mothers’ education level and morbidity with a deviance of 8247.1 on 6 df is significantly different from the full model ($\chi ^2_{0.05;6}=12.59$); thus rejecting the null model of no association between mother’s education level and morbidity. The additive model for wealth quintile and morbidity with a deviance of 6491.3 on 8 df is significantly different from the full model ($\chi ^2_{0.05;8}=15.51$); hence, rejecting the null model of no association between wealth quintile and morbidity. The $\star$ in the tables indicates not statistically significant at $\alpha =0.05$ level of significance.

Furthermore, the multiplicative models in Tables 2 were fitted to explore the suggested association between geopolitical zones and morbidity by narrowing the comparison of under five morbidity down to the states in each of the six geopolitical zones. Previous studies like^24^ found the existence of significant regional and location differentials in the susceptibility to morbidity (particularly, diarrhoea and ARI among children under 5 years old in Uganda). We did not include ARI due to the non-availability of the data on a state-by-state basis. However, as we shall see children under the age of five in Nigeria are generally more likely to have diarrhoea and fever than ARI and this concurs with^25^ who found a decreasing pattern in the development of ARI symptoms among under five children over the decades between regions in Nigeria. We discuss the results of the entire analysis as presented in Tables 1-2 in the sequel.

**Table 1:** Results of the Poisson regression model fitted to describe the relationship between childhood morbidity in Nigeria and various background characteristics.

Coefficients	Estimate (log odds)	Odds/OR (95% C.I)	Std. error	*z* value	$P-$value
Age (months) and morbidity					
Intercept	6.44889	–	0.03978	162.123	$< 2.0 \times 10^{-16}$
< 6	− 1.98298	0.138 (0.110,0.172)	0.11435	− 17.341	$< 2.0\times 10^{-16}$
6–11	− 0.24030	0.786 (0.699,0.884)	0.05995	− 4.008	$6.1\times 10^{-5}$
12–23	− 0.69632	0.498 (0.435,0.571)	0.06897	− 10.096	$< 2.0\times 10^{-16}$
24–34	− 1.69530	0.184 (0.151,0.224)	0.10101	− 16.784	$< 2.0\times 10^{-16}$
36–47	0.36894	1.446 (1.307,1.601)	0.05173	7.132	$9.9\times 10^{-13}$
Diarrhoea	− 0.41820	0.658 (0.582,0.745)	0.06314	− 6.624	$3.5\times 10^{-11}$
Fever	0.24443	1.277 (1.151,1.417)	0.05312	4.602	$4.1\times 10^{-6}$
$< 6$ : diarrhoea	0.75632	2.130 (1.576,2.880)	0.15387	4.915	$8.8\times 10^{-7}$
6–11: diarrhoea	1.46662	4.335 (3.692,5.089)	0.08187	17.913	$< 2.0\times 10^{-16}$
12–23: diarrhoea	0.95163	2.590 (2.150,3.120)	0.09499	10.018	$< 2.0\times 10^{-16}$
24–34: diarrhoea	0.59909	1.820 (1.382,2.399)	0.14072	4.257	$2.0\times 10^{-5}$
36–47: diarrhoea	0.74715	2.111 (1.817,2.453)	0.07660	9.754	$< 2.0\times 10^{-16}$
$< 6$ : Fever	0.63677	1.890 (1.442,2.478)	0.13812	4.610	$4.0\times 10^{-6}$
6–11: fever	1.05686	2.877 (2.492,3.322)	0.07336	14.407	$< 2.0\times 10^{-16}$
12–23: fever	1.12747	3.088 (2.627,3.630)	0.08247	13.671	$< 2.0\times 10^{-16}$
24–34: fever	− 0.04926	0.952 (0.729,1.243)	0.13614	− 0.362	0.717$^{\star }$
36–47: fever	0.27792	1.320 (1.157,1.507)	0.06756	4.114	$3.8\times 10^{-5}$
Child gender and morbidity					
Intercept	6.03309	–	0.04897	123.20	$< 2.0\times 10^{-16}$
Female	2.18354	8.878 (8.023,9.823)	0.05165	42.27	$< 2.0\times 10^{-16}$
Diarrhoea	2.20015	9.026 (8.158,9.987)	0.05161	42.63	$< 2.0\times 10^{-16}$
Fever	1.56531	4.784 (4.305,5.317)	0.05385	29.07	$< 2.0\times 10^{-16}$
Female: diarrhoea	− 2.83863	0.059 (0.052,0.066)	0.05870	− 48.36	$< 2.0\times 10^{-16}$
Female: fever	− 3.79801	0.022 (0.019,0.026)	0.07542	− 50.36	$< 2.0\times 10^{-16}$
Residence and morbidity					
Intercept	5.47646	–	0.06468	84.66	$< 2.0\times 10^{-16}$
Urban	2.25063	9.494 (8.309,10.847)	0.06801	33.09	$< 2.0\times 10^{-16}$
Diarrhoea	3.07937	21.745 (19.100,24.755)	0.06616	46.55	$< 2.0\times 10^{-16}$
Fever	1.58915	4.900 (4.263,5.631)	0.07098	22.39	$< 2.0\times 10^{-16}$
Urban: diarrhoea	− 2.87662	0.056 (0.049,0.065)	0.07195	− 39.98	$< 2.0\times 10^{-16}$
Urban: fever	− 2.96187	0.052 (0.044,0.061)	0.08496	− 34.86	$< 2.0\times 10^{-16}$
Zone and morbidity					
Intercept	6.18621	–	0.04536	136.377	$< 2.0\times 10^{-16}$
North Central	− 2.17888	0.113 (0.086,0.150)	0.14227	− 15.316	$< 2.0\times 10^{-16}$
Northeast	0.43918	1.551 (1.384,1.739)	0.05817	7.550	$4.3\times 10^{-14}$
Southeast	− 0.05281	0.949 (0.835,1.077)	0.06501	− 0.812	0.417$^{\star }$
South-South	1.39398	4.031 (3.650,4.452)	0.05068	27.508	$< 2.0\times 10^{-16}$
Southwest	1.04218	2.835 (2.557,3.144)	0.05276	19.754	$< 2.0\times 10^{-16}$
Diarrhoea	− 1.05041	0.350 (0.294,0.417)	0.08911	− 11.788	$< 2.0\times 10^{-16}$
Fever	1.13034	3.097 (2.796,3.430)	0.05217	21.665	$< 2.0\times 10^{-16}$
North Central: diarrhoea	1.23273	3.431 (2.304,5.109)	0.20316	6.068	$1.3\times 10^{-9}$
Northeast: diarrhoea	0.99028	2.692 (2.198,3.296)	0.10332	9.585	$< 2.0\times 10^{-16}$
Southeast: diarrhoea	− 1.55663	0.211 (0.143,0.311)	0.19880	− 7.830	$4.8\times 10^{-15}$
South-South: diarrhoea	− 0.64368	0.525 (0.427,0.647)	0.10596	− 6.075	$1.2\times 10^{-9}$
Southwest: diarrhoea	− 0.80270	0.448 (0.357,0.562)	0.11531	− 6.962	$3.3\times 10^{-12}$
North Central: fever	− 0.15407	0.857 (0.618,1.188)	0.16659	− 0.925	0.355$^{\star }$
Northeast: fever	0.26355	1.302 (1.143,1.482)	0.06616	3.983	$6.7\times 10^{-5}$
Southeast: fever	− 3.31249	0.036 (0.027,0.049)	0.15531	− 21.328	$< 2.0\times 10^{-16}$
South-South: fever	− 2.24438	0.106 (0.093,0.121)	0.06919	− 32.436	$< 2.0\times 10^{-16}$
Southwest: fever	− 3.08061	0.046 (0.038,0.055)	0.09247	− 33.316	$< 2.0\times 10^{-16}$
Education of child’s mother and morbidity					
Intercept	6.07764	–	0.04789	126.91	$< 2.0 \times 10^{-16}$
Primary	2.21190	9.133 (8.273,10.082)	0.05045	43.85	$< 2.0\times 10^{-16}$
Secondary	1.64371	5.174 (4.670,5.733)	0.05231	31.42	$< 2.0\times 10^{-16}$
> Secondary	− 1.05376	0.349 (0.290,0.419)	0.09419	− 11.19	$< 2.0\times 10^{-16}$
Diarrhoea	0.75215	2.122 (1.893,2.377)	0.05809	12.95	$< 2.0\times 10^{-16}$
Fever	0.95851	2.608 (2.335,2.912)	0.05633	17.02	$< 2.0\times 10^{-16}$
Primary: diarrhoea	− 5.74585	0.003 (0.002,0.005)	0.20165	− 28.49	$< 2.0\times 10^{-16}$
Secondary: diarrhoea	− 2.57635	0.076 (0.065,0.089)	0.08103	− 31.80	$< 2.0\times 10^{-16}$
> Secondary: diarrhoea	− 0.65207	0.521 (0.407,0.667)	0.12612	− 5.17	$2.3\times 10^{-7}$
Primary: fever	− 2.84945	0.058 (0.050,0.067)	0.07133	− 39.95	$< 2.0\times 10^{-16}$
Secondary: fever	− 3.38655	0.034 (0.028,0.041)	0.09296	− 36.43	$< 2.0\times 10^{-16}$
> Secondary: fever	1.60947	5.000 (4.100,6.098)	0.10127	15.89	$< 2.0\times 10^{-16}$
Wealth quintile and morbidity					
Intercept	7.56528	–	0.02276	332.356	$< 2.0\times 10^{-16}$
Fourth	− 2.22774	0.108 (0.093,0.124)	0.07298	− 30.526	$< 2.0\times 10^{-16}$
Lowest	− 1.94126	0.144 (0.127,0.163)	0.06425	− 30.213	$< 2.0\times 10^{-16}$
Middle	− 0.44726	0.639 (0.595,0.687)	0.03645	− 12.271	$< 2.0\times 10^{-16}$
Second	0.10934	1.116 (1.049,1.186)	0.03135	3.488	0.0005
Diarrhoea	− 1.78145	0.168 (0.150,0.189)	0.05996	− 29.711	$< 2.0\times 10^{-16}$
Fever	− 2.84678	0.058 (0.048,0.070)	0.09719	− 29.290	$< 2.0\times 10^{-16}$
Fourth: diarrhoea	3.05730	21.270 (17.528,25.811)	0.09872	30.970	$< 2.0\times 10^{-16}$
Lowest: diarrhoea	3.18563	24.183 (20.274,28.845)	0.08995	35.414	$< 2.0\times 10^{-16}$
Middle: diarrhoea	− 1.05990	0.346 (0.266,0.452)	0.13526	− 7.836	$4.6\times 10^{-15}$
Second: diarrhoea	0.40945	1.506 (1.296,1.750)	0.07675	5.335	$9.5\times 10^{-8}$
Fourth: fever	4.16339	64.289 (50.352,82.084)	0.12467	33.395	$< 2.0\times 10^{-16}$
Lowest: fever	4.18026	65.383 (51.846,82.454)	0.11836	35.319	$< 2.0\times 10^{-16}$
Middle: fever	3.04863	21.086 (17.181,25.879)	0.10450	29.175	$< 2.0\times 10^{-16}$
Second: fever	0.14889	1.161 (0.900,1.496)	0.12964	1.148	0.2508$^{\star }$

**Table 2:** Results of the Poisson regression model fitted to describe the relationship between childhood morbidity in Nigeria and states by zones.

Coefficients	Estimate (log odds)	Odds/OR (95% C.I)	Std. error	*z* value	$P-$value
North central states and morbidity					
Intercept	3.89182	–	0.14286	27.243	$< 2.0\times 10^{-16}$
Benue	− 1.05861	0.347 (0.200,0.602)	0.28148	− 3.761	0.000169
Kogi	0.81771	2.265 (1.619,3.170)	0.17151	4.768	$1.8\times 10^{-6}$
Kwara	0.72330	2.061 (1.465,2.899)	0.17410	4.155	$3.2\times 10^{-5}$
Nasarawa	− 0.06318	0.939 (0.628,1.404)	0.20530	− 0.308	0.758278$^{\star }$
Niger	− 0.63372	0.531 (0.330,0.854)	0.24263	− 2.612	0.009004
Plateau	0.15123	1.163 (0.794,1.704)	0.19481	0.776	0.437579$^{\star }$
Diarrhoea	− 0.17825	0.837 (0.553,1.267)	0.21166	− 0.842	0.399699$^{\star }$
Benue: diarrhoea	1.21624	3.374 (1.690,6.737)	0.35278	3.448	0.000566
Kogi: diarrhoea	− 1.23545	0.291 (0.161,0.525)	0.30140	− 4.099	$4.1\times 10^{-5}$
Kwara: diarrhoea	1.38617	4.000 (2.498,6.403)	0.24012	5.773	$7.8\times 10^{-9}$
Nasarawa: diarrhoea	1.63281	5.118 (3.029,8.648)	0.26761	6.102	$1.0\times 10^{-9}$
Niger: diarrhoea	1.57411	4.826 (2.657,8.768)	0.30460	5.168	$2.3\times 10^{-7}$
Plateau: diarrhoea	0.45269	1.573 (0.917,2.696)	0.27509	1.646	0.099851$^{\star }$
Northeast states and morbidity					
Intercept	5.29330	−	0.07089	74.671	$< 2.0\times 10^{-16}$
Bauchi	− 0.96257	0.382 (0.293,0.497)	0.13484	− 7.138	$9.4\times 10^{-13}$
Borno	1.18674	3.276 (2.795,3.840)	0.08099	14.653	$< 2.0\times 10^{-16}$
Gombe	0.81372	2.256 (1.909,2.666)	0.08516	9.555	$< 2.0\times 10^{-16}$
Taraba	− 0.08930	0.915 (0.748,1.118)	0.10257	− 0.871	0.384$^{\star }$
Yobe	− 0.67818	0.508 (0.399,0.645)	0.12217	− 5.551	$2.8\times 10^{-8}$
Diarrhoea	0.15343	1.166 (0.965,1.409)	0.09662	1.588	0.112$^{\star }$
Bauchi: diarrhoea	0.90946	2.483 (1.799,3.427)	0.16443	5.531	$3.1\times 10^{-8}$
Borno: diarrhoea	− 1.35536	0.258 (0.201,0.330)	0.12638	− 10.725	$< 2.0\times 10^{-16}$
Gombe: diarrhoea	− 1.19786	0.302 (0.232,0.392)	0.13376	− 8.955	$< 2.0\times 10^{-16}$
Taraba: diarrhoea	0.85115	2.342 (1.816,3.021)	0.12978	6.559	$5.4\times 10^{-11}$
Yobe : diarrhoea	1.15303	3.168 (2.370,4.234)	0.14805	7.788	$6.7\times 10^{-15}$
Northwest states and morbidity					
Intercept	6.41673	−	0.04042	158.741	$< 2.0\times 10^{-16}$
Jigawa	− 0.34831	0.706 (0.624,0.798)	0.06284	− 5.543	$2.9\times 10^{-8}$
Kaduna	− 0.92367	0.397 (0.342,0.461)	0.07582	− 12.182	$< 2.0\times 10^{-16}$
Kano	− 0.14953	0.861 (0.766,0.967)	0.05943	− 2.516	0.0119
Kastina	− 0.93194	0.394 (0.339,0.457)	0.07605	− 12.254	$< 2.0\times 10^{-16}$
Kebbi	0.04161	1.042 (0.933,1.165)	0.05658	0.735	0.4621$^{\star }$
Sokoto	− 0.35761	0.699 (0.618,0.791)	0.06301	− 5.675	$1.3\times 10^{-8}$
Diarrhoea	− 2.61007	0.074 (0.054,0.100)	0.15445	− 16.899	$< 2.0\times 10^{-16}$
Jigawa: diarrhoea	2.22522	9.256 (6.607,12.965)	0.17197	12.940	$< 2.0\times 10^{-16}$
Kaduna: diarrhoea	3.08572	21.883 (15.537,30.821)	0.17473	17.660	$< 2.0\times 10^{-16}$
Kano: diarrhoea	0.91758	2.503 (1.725,3.632)	0.18990	4.832	$1.3\times 10^{-6}$
Kastina: diarrhoea	2.75289	15.688 (11.072,22.227)	0.17777	15.486	$< 2.0\times 10^{-16}$
Kebbi: diarrhoea	1.21433	3.368 (2.375,4.776)	0.17819	6.815	$9.4\times 10^{-12}$
Sokoto : diarrhoea	1.62612	5.084 (3.572,7.237)	0.18012	9.028	$< 2.0\times 10^{-16}$
Southeast states and morbidity					
Intercept	3.40120	–	0.18257	18.629	$< 2.0\times 10^{-16}$
Anambra	− 0.91629	0.400 (0.205,0.781)	0.34157	− 2.683	0.0073
Ebonyi	1.51878	4.567 (3.076,6.779)	0.20158	7.535	$4.9\times 10^{-14}$
Enugu	0.03279	1.033 (0.626,1.707)	0.25611	0.128	0.8981$^{\star }$
Imo	2.10413	8.200 (5.613,11.979)	0.19339	10.880	$< 2.0\times 10^{-16}$
Diarrhoea	0.98083	2.667 (1.753,4.057)	0.21409	4.581	$4.6\times 10^{-6}$
Anambra: diarrhoea	0.69315	2.000 (0.949,4.216)	0.38052	1.822	0.0685$^{\star }$
Ebonyi: diarrhoea	− 3.01044	0.049 (0.026,0.094)	0.32968	− 9.131	$< 2.0\times 10^{-16}$
Enugu: diarrhoea	0.70318	2.020 (1.144,3.566)	0.28996	2.425	0.0153
Imo: diarrhoea	− 2.49718	0.082 (0.049,0.137)	0.26157	− 9.547	$< 2.0\times 10^{-16}$
South-South states and morbidity					
Intercept	3.6636	–	0.1601	22.879	$< 2.0\times 10^{-16}$
Akwa Ibom	1.5294	4.615 (3.265,6.524)	0.1766	8.659	$< 2.0\times 10^{-16}$
Cross River	− 0.6190	0.538 (0.317,0.915)	0.2707	− 2.287	0.022190
Delta	− 2.5649	0.077 (0.024,0.249)	0.5991	− 4.281	$1.8\times 10^{-5}$
Edo	0.2485	1.282 (0.844,1.949)	0.2136	1.163	0.244828$^{\star }$
Rivers	− 1.0986	0.333 (0.178,0.624)	0.3203	− 3.430	0.000603
Diarrhoea	− 0.5725	0.564 (0.335,0.951)	0.2666	− 2.147	0.031779
Akwa Ibom: diarrhoea	− 1.2192	0.295 (0.154,0.566)	0.3316	− 3.676	0.000237
Cross River: diarrhoea	1.8047	6.078 (2.977,12.408)	0.3641	4.956	$7.2\times 10^{-7}$
Delta: diarrhoea	2.3071	10.045 (2.646,38.133)	0.6806	3.390	0.000700
Edo: diarrhoea	2.5354	12.621 (6.923,23.010)	0.3064	8.274	$< 2.0\times 10^{-16}$
Rivers: diarrhoea	2.3514	10.500 (4.782,23.057)	0.4013	5.860	$4.6\times 10^{-9}$
Southwest states and morbidity					
Intercept	3.332	−	$1.8\times 10^{-1}$	17.632	$< 2.0\times 10^{-16}$
Ekiti	$6.3\times 10^{-1}$	1.893 (1.198,2.992)	$2.3\times 10^{-1}$	2.731	0.006310
Ogun	1.243	3.466 (2.276,5.277)	$2.1\times 10^{-1}$	5.792	$6.9\times 10^{-9}$
Ondo	$7.1\times 10^{-1}$	2.036 (1.295,3.200)	$2.3\times 10^{-1}$	3.080	0.002068
Osun	$-6.3\times 10^{-16}$	1.000 (0.592,1.689)	$2.6\times 10^{-1}$	0.000	1.000000$^{\star }$
Oyo	− 1.723	0.179 (0.069,0.462)	$4.8\times 10^{-1}$	− 3.548	0.000388
Diarrhoea	$-3.6\times 10^{-2}$	0.964 (0.568,1.636)	$2.6\times 10^{-1}$	− 0.135	0.892744$^{\star }$
Ekiti: diarrhoea	$-2.1\times 10^{-2}$	0.978 (0.508,1.883)	$3.3\times 10^{-1}$	− 0.066	0.947733$^{\star }$
Ogun: diarrhoea	$-2.6\times 10^{-1}$	0.770 (0.418,1.417)	$3.1\times 10^{-1}$	− 0.840	0.400676$^{\star }$
Ondo: diarrhoea	$-1.7\times 10^{-1}$	0.837 (0.434,1.613)	$3.3\times 10^{-1}$	− 0.532	0.594780$^{\star }$
Osun: diarrhoea	$7.9\times 10^{-1}$	2.222 (1.111,4.445)	$3.5\times 10^{-1}$	2.257	0.023988
Oyo: diarrhoea	2.397	10.990 (3.814,31.671)	$5.4\times 10^{-1}$	4.439	$9.0\times 10^{-6}$

### Age (months) versus morbidity

The likelihood of experiencing ARI among children under the age of five is significantly lower by about 0.14 times in children under 6 months old, followed by children under 24–34 months by about 0.18 times, children under 12–23 months by about 0.5 times, and children under 6–11 months by 0.79 times. The likelihood is significantly higher among children under 36–47 months by about 1.45 times compared to children under 48–59 months; our result is opposite of the findings in Kandala et al.^26^ who reported that the risk of developing diarrhoea, cough, and fever by under five children in Nigeria increased in the first 6–8 months and then decreased slowly. Geberetsadik and Berhane^27^ found that children aged 48–59 months are about 0.5 times less likely to observe ARI compared to children under 6 months old in Ethiopia. In general, children under 48–59 months tend to experience fever by about 1.28 times more and diarrhoea by about 0.66 times less than ARI. The odds of experiencing diarrhoea by a child less than 6 months old is about 2.1 times more than that of experiencing ARI by a child in that age group compared to a child under 48–59 months. The odds of experiencing diarrhoea by a child aged between 6–11 months old is about 4.3 times more than that of experiencing ARI by a child in that age group compared to a child under 48–59 months. The odds of experiencing diarrhoea by a child aged between 12–23 months old is about 2.6 times more than that of experiencing ARI by a child in that age group compared to a child under 48–59 months. The odds of experiencing diarrhoea by a child aged between 24–34 months old is about 1.8 times more than that of experiencing ARI by a child in that age group compared to a child under 48–59 months. The odds of experiencing diarrhoea by a child aged between 36–47 months old is about 2.1 times more than that of experiencing ARI by a child in that age group compared to a child under 48–59 months. The odds of experiencing fever by a child under 6 months old is about 1.9 times more than that of experiencing ARI by a child in that age group compared to a child under 48–59 months. The odds of experiencing fever by a child aged between 6–11 months old is about 2.9 times more than that of experiencing ARI by a child in that age group compared to a child under 48–59 months. The odds of experiencing fever by a child aged between 12–23 months old is about 3.1 times more than that of experiencing ARI by a child in that age group compared to a child under 48–59 months. The odds of experiencing fever by a child aged between 24–34 months old is the same as that of experiencing ARI by a child of the same age group compared to a child under 48–59 months. The odds of experiencing fever by a child aged 36–47 months old is about 1.3 times more than that of experiencing ARI by a child in that age group compared to a child under 48–59 months.

### Gender and location versus morbidity

Unlike^28^ who found that ARI affects more male children under the age of five than their female counterparts but not significantly in the Kancheepuram district of South India, Khalek and Abdel-Salam^29^ found that male children are more susceptible to ARI than female children in upper Egypt, and Savitha and Gopalakrishnan^30^ found that male children are more likely to indicate ARI than female children in rural areas of Tamil Nadu in India. However, using the 2013 NDHS data,^31^ did not find any statistically significant association between ARI and gender. Our results show that ARI is about 9 times more prevalent among female children aged less than 5 years old than male children aged less than 5 years old. The odds of experiencing diarrhoea among male children under 5 years old is about 9 times more than that of experiencing ARI among them. The odds of experiencing fever among male children is about 5 times more than that of experiencing ARI among them. Children domiciled in urban areas are about 9.5 times more likely to experience ARI than those in rural areas, contradicting^28^ who reported that ARI is more prevalent among children in rural areas than children in urban areas of Kancheepuram district in South India and^32^ who identified that ARI is less likely to affect children in urban areas compared to children in rural areas in Rwanda. Children in rural areas are about 22 times more likely to experience diarrhoea than ARI and about 5 times more likely to experience fever than ARI. This resonates with^26^ who found that under five children in Nigeria residing in urban areas are at lower risk of fever than their counterparts in rural areas. However, our findings partially contradict^33^, who reported that under five children residing in urban areas of the Democratic Republic of Congo are less likely to experience fever and ARI. The odds of experiencing diarrhoea (fever) among female children is about 0.06 (0.02) times less than that of experiencing ARI compared to male children. This is similar to what^34^ found in Nigeria concerning diarrhoea prevalence between male and female under five children and what^35^ found concerning the prevalence of diarrhoea between male and female under five children in Sudan. The odds of having diarrhoea (fever) among children living in urban areas is about 0.06 (0.05) less than that of having ARI in children dwelling in urban areas compared to children living in rural areas. This contradicts^35^, who reported that children in urban areas of Sudan are more likely to suffer from diarrhoea compared to children in rural settings. But our result is consistent with^36^ who used the 2013 NDHS data and found that diarrhoea is more likely to be observed among under five children in rural areas than children in urban areas.

### Zone versus morbidity

More recently,^37^ analysed the 2018 NDHS data. They reported that under five children are more likely to suffer morbidity in the Northeast than in any other geopolitical zone in Nigeria. This paper shows that ARI is about 0.113 times less likely to manifest among children under 5 years old in the North Central than in the Northwest. There are no significant differences between the odds of experiencing ARI among Southeastern and Northwestern children. Children in the Northeast are about 1.6 times more likely to experience ARI than those in the Northwest. Children in the Southwest are about 2.84 times more likely to experience ARI than children in the Northwest, and children in the South-South are about 4.03 times more likely to experience ARI than children in the Northwest. Children in the Northwest are about 3.1 times more likely to experience fever than ARI. They are about 0.35 less likely to have diarrhoea than ARI. The odds of experiencing diarrhoea among children under 5 years old in the North Central zone is about 3.4 times more than that of experiencing ARI compared to children in the Northwestern zone. The odds of experiencing diarrhoea by under five children in the Northeastern zone is about 2.7 times more than that of experiencing ARI compared to children in the Northwestern zone. The odds of experiencing diarrhoea among under five children in the Southeastern zone is about 0.2 times less than that of experiencing ARI compared to children in the Northwestern zone. The odds of experiencing diarrhoea among under five children in the South-Southern zone is about 0.5 times less than that of experiencing ARI compared to children in the Northwestern zone. The odds of experiencing diarrhoea among under five children in the Southwestern zone is about 0.4 times less than that of experiencing ARI compared to children in the Northwestern zone. The odds of experiencing fever among under five children in the North Central zone is about 0.9 times less than that of experiencing ARI compared to children in the Northwestern zone. The odds of experiencing fever among under five children in the Northeastern zone is about 1.3 times more than that of experiencing ARI compared to children in the Northwestern zone. The odds of experiencing fever among under five children in the Southeastern zone is about 0.04 times less than that of experiencing ARI compared to children in the Northwestern zone. The odds of experiencing fever among under five children in the South-Southern zone is about 0.1 times less than that of experiencing ARI compared to children in the Northwestern zone. The odds of experiencing fever among under five children in the Southwestern zone is about 0.05 times less than that of experiencing ARI compared to children in the Northwestern zone. Some of the findings here are similar to those of^38^. However, the majority of our results are in discrepancy with those in Kandala et al.^38^ who found with the 1999 and 2003 NDHS data that there is a higher prevalence of diarrhoea, cough, and fever in the Northern and Eastern states of Nigeria than in the Western and Southern states of Nigeria. Berde et al.^39^ analyzed the 2013 NDHS data and reported that under five children from every other geopolitical zone have a higher risk of diarrhoea than their mates in the South-South. The relationship between the mortality and morbidity of under five children is well-known and documented. Going by this fact, we can find contradictions from Kandala et al.^38^ and Adedini et al.^11^ who reported that under five children from the Yoruba ethnic group (Southwestern Nigeria) possess the lowest mortality risk, followed by their mates from the Igbo ethnic group (Southeastern Nigeria) and then children from the Hausa/Fulani/Kanuri ethnic groups (Northern Nigeria). Similarly,^40^ reported that under five children whose mothers are from the Hausa/Fulani/Kanuri tribe have the highest mortality risk while under five children whose mothers are from the Igbo tribe have lower mortality risk. However, children whose mothers are from the Yoruba tribe have the lowest mortality risk. The findings in Antai^40^ and Adedini et al.^11^ largely concur with our findings. The obvious variations between the results in this paper and those of Kandala et al.^38^ could be explained by the large time lag between the 1999/2003 NDHS data and the latest 2018 NHDS data (which is more than a decade). So, one should expect some significant changes, perhaps due to medical interventions, increased awareness of children’s morbidity, sociocultural changes, and economic downturn.

### Mothers education versus morbidity

Contrary to Bbaale^24^ and Adesanya and Chiao^31^, we found that under five children whose mothers had more than secondary education are about 0.35 times less likely to have ARI compared to children whose mothers had no education. Children whose mothers have primary (secondary) education are about 9.1 (5.2) more likely to suffer from ARI than children whose mothers did not have any formal education. Children whose mothers have no education are about 2.1 (respectively, 2.6) times more likely to suffer from diarrhoea (respectively, fever) compared to ARI. This finding is similar to^41^ who used the 2013 NDHS data and reported that children whose mothers have no education are more likely to experience diarrhoea. Mohammed et al.^42^ found through the analysis of a community-based cross-sectional study in the Arba-Minch district, Southern Ethiopia, that diarrhoea is more prevalent among under five children whose mothers have no formal education. The odds of having diarrhoea among under five children whose mothers have primary education is about 0.003 times less than that of having ARI compared to children whose mothers have no education. The odds of having diarrhoea among under five children whose mothers have secondary education is about 0.08 times less than that of having ARI compared to children whose mothers have no education. The odds of having diarrhoea among under five children whose mothers have more than secondary education is about 0.5 times less than that of having ARI compared to children whose mothers have no education. The odds of having fever among under five children whose mothers have primary education is about 0.06 times less than that of having ARI compared to children whose mothers have no education. The odds of having fever among under five children whose mothers have secondary education is about 0.03 times less than that of having ARI compared to children whose mothers have no education. The odds of having fever among under five children whose mothers have more than secondary education is about 5 times more than that of having ARI compared to children whose mothers have no education.

### Wealth quintile versus morbidity

Contrary to the study in Kancheepuram district of South India^31,28^ used the 2013 NDHS data to find that under five children who fall under the category of fourth, middle, and lowest wealth quintiles are generally about 0.11, 0.64, and 0.14 times less likely to suffer from ARI compared to children in the fifth wealth quintile. Diarrhoea and fever are about 0.17 and 0.06 times less likely to manifest among children in the fifth wealth quintile. Our results for fever are consistent with^43,44^ and^45^. Under five children in the fourth, second and lowest wealth quintiles are about 21.27, 1.51, and 24.18 times more likely to have diarrhoea than ARI compared to children in the fifth wealth quintile. Similarly, based on the 2013 NDHS data,^41^ reported that diarrhoea is more common among children in the lowest wealth quintile. Under five children in the middle wealth quintile are about 0.35 times less likely to have diarrhoea than ARI compared to children in the fifth wealth quintile. Under five children in the fourth, middle, and lowest wealth quintiles are about 64.29, 21.09, and 65.38 times more likely to experience fever than ARI compared to children in the fifth wealth quintile. However, the probabilities of experiencing fever and ARI among under five children in the second and fifth wealth quintiles appear to be equal. Kandala et al.^26^, who also noticed inconsistencies in the results pertaining to the socio-economic status like in this paper, attributed that the problem of category misclassification could be one of the major limitations in NDHS surveys, where the socioeconomic status of the household was only determined by the response of a female member of that household instead of an independent assessment based on say, family income, assets and employment.

### North Central versus morbidity

Under five children in Benue state are about 0.35 times less likely to experience fever than children in the Federal Capital Territory (FCT). Under five children in Niger state are about 0.53 times less likely to experience fever than children in the FCT whereas under five children in Kwara state are about 2.1 times more likely to experience fever compared to children in the FCT. Under five children in Kogi state are about 2.3 times more likely to experience fever than their counterparts in the FCT. The odds of experiencing fever by a child under the age of five are the same in Nasarawa and Plateau states compared to the FCT. The odds of experiencing diarrhoea by a child less than 5 years old is not significantly different from that of experiencing fever in the FCT. The odds of having diarrhoea in a child under 5 years old in Benue state is about 3.4 times more than that of having a fever in a child in Benue state compared to the FCT. The odds of having diarrhoea in a child under 5 years old in Kogi state is about 0.3 times less than that of having a fever in a child in Kogi state compared to the FCT. The odds of having diarrhoea among under five children in Kwara state is about 4 times more than that of having a fever in Kwara state compared to the FCT. The odds of having diarrhoea in under five children in Nasarawa state is about 5.1 times more than that of having a fever in Nasarawa state compared to the FCT. The odds of having diarrhoea in under five children in Niger state is about 4.8 times more than that of having fever in Niger state compared to the FCT. The odds of experiencing diarrhoea in under five children in Plateau state is about 1.6 times more than that of experiencing fever in Plateau state compared to the FCT.

### Northeast versus morbidity

Children less than 5 years old are about 0.38 and 0.51 times less likely to have fever in Bauchi and Yobe states. They are about 2.3 and 3.3 times more likely to have fever in Gombe and Borno states. However, their odds of having a fever are equivalent in Taraba and Adamawa states. The odds of having diarrhoea in a child under 5 years old in Bauchi state is about 2.5 times more than that of having a fever in a child in Bauchi state compared to Adamawa state. The odds of having diarrhoea among under five children are not significantly different from that of having a fever in Adamawa state. The odds of having diarrhoea among under five children in Borno state is about 0.3 times less than that of having a fever in Borno state compared to Adamawa state. The odds of having diarrhoea among under five children in Gombe state is about 0.3 times less than that of having a fever in Gombe state compared to Adamawa state. The odds of having diarrhoea among under five children in Taraba state is about 2.3 times more than that of having a fever in Taraba state compared to Adamawa state. The odds of having diarrhoea among under five children in Yobe State is about 3.2 times more than having a fever in Yobe State compared to Adamawa State.

### Northwest versus morbidity

The odds of observing fever in children less than 5 years old in Kebbi state is not significantly different from that of observing it in Zamfara state. However, the odds are significantly less by about 0.4, 0.4, 0.7, 0.7, and 0.9 times in Kastina, Kaduna, Sokoto, Jigawa, and Kano states, respectively, compared to Zamfara state. Under five children are about 0.07 times less likely to experience diarrhoea than fever in Zamfara state. The odds of having diarrhoea in under five children in Jigawa state is about 9.3 times more than that of having fever in Jigawa state compared to Zamfara state. The odds of having diarrhoea among under five children in Kaduna state is about 22 times more than that of having a fever in Kaduna state compared to Zamfara state. The odds of having diarrhoea among under five children in Kano state is about 2.5 times more than that of having a fever in Kano state compared to Zamfara state. The odds of having diarrhoea among under five children in Kastina state is about 16 times more than that of having a fever in Kastina state compared to Zamfara state. The odds of experiencing diarrhoea in under five children in Kebbi state is about 3.4 times more than that of experiencing fever in Kebbi state compared to Zamfara state. The odds of observing diarrhoea among under five children in Sokoto state is about 5.1 times more than that of observing fever in Sokoto state compared to Zamfara state.

### Southeast versus morbidity

Children less than 5 years old are about 0.4 times less likely to experience fever in Anambra state than in Abia state. But they are about 4.6 (respectively, 8.2) times more likely to have a fever in Ebonyi (respectively, Imo) state than in Abia state. However, the odds of observing fever among under five children in Enugu state is the same as that of observing it in Abia state. Children less than 5 years old are about 2.7 times more likely to have diarrhoea than fever in Abia state. The odds of having diarrhoea among under five children in Anambra state is about 2 times more than that of having a fever in Anambra state compared to Abia state. The odds of having diarrhoea in under five children in Ebonyi state is about 0.05 times less than that of having fever in Ebonyi state compared to Abia state. The odds of having diarrhoea among under five children in Enugu state is about 2.02 times more than that of having fever in Enugu state compared to Abia state. The odds of having diarrhoea among under five children in Imo state is about 0.08 times less than that of having fever in Imo state compared to Abia state.

### South-South versus morbidity

The odds of experiencing fever among children under 5 years old in Edo state is the same as that of experiencing it in Bayelsa state. However, the odds of observing fever among these children decreases by about 0.08, 0.3 and 0.5 times in Delta, Rivers and Cross Rivers states, respectively. It increases by about 5 times in Akwa Ibom state compared to Bayelsa state. Diarrhoea is about 0.6 times less likely to occur among children under 5 years old than fever in Bayelsa state. The odds of having diarrhoea in a child under 5 years old in Akwa Ibom state is about 0.3 times less than that of having a fever in a child in Akwa Ibom state compared to Bayelsa state. The odds of experiencing diarrhoea in a child under 5 years old in Cross River state is about 6.1 times more than that of experiencing fever by a child in Cross River state compared to Bayelsa state. The odds of having diarrhoea in a child under 5 years old in Delta state is about 10 times more than that of having a fever in a child in Delta state compared to Bayelsa state. The odds of an under five child observing diarrhoea in Edo state is about 13 times more than that of observing fever in Edo state compared to Bayelsa state. The odds of experiencing diarrhoea in a child under 5 years old in Rivers state is about 11 times more than that of experiencing fever in Rivers state compared to Bayelsa state.

### Southwest versus morbidity

The odds of experiencing fever in a child under 5 years old in Osun state is the same as that of experiencing it in Lagos state. However, the odds of fever in children under 5 years old decreases by about 0.2 times in Oyo state. It increases by about 1.9, 2, and 3.5 times in Ekiti, Ondo and Ogun states, respectively, compared to Lagos state. There are no significant differences between the odds of observing diarrhoea and fever among under five children in Lagos state. The odds of observing diarrhoea among under five children in Ekiti, Ogun, Ondo, and Osun states are the same as that of observing fever among under five children in those states compared to Lagos state. The odds of experiencing diarrhoea in a child under 5 years old in Oyo state is about 11 times more than that of experiencing fever in Oyo state compared to Lagos state.

## Conclusions

In Nigeria, the under five children residing in rural areas are more susceptible to fever and diarrhoea than ARI, while those in the urban regions are more susceptible to ARI than fever and diarrhoea. We have also found that children less than 3 years old are at a lower risk of ARI whereas, in Uganda, children under 2 years old were found to be more susceptible to ARI^46^. Children between the ages of three to 4 years old have a higher risk of ARI than children above 4 years old. Children 4 years old and above are at a higher risk of fever but lower risk of diarrhoea compared to ARI. Children less than 6 months old, 6 months old to less than 1 year old, 1 year old to less than 2 years old and 3 years old to less than 4 years old are at a higher risk of diarrhoea and fever than ARI compared to children 4 years old and above. However, children between 2 years old and 3 years old have a higher risk of diarrhoea but a similar risk of fever compared to children 4 years old and above. Our result is consistent with^47^ who found that the prevalence rate of diarrhoea was highest among children under 1 year old in rural areas of Akoko North, Ondo state, Nigeria and Oni et al.^48^ who found that diarrhoea incidence decreases with children’s age in a low-income traditional area of Ilorin, Kwara state, Nigeria.

We found evidence that female under five children are at a higher risk of ARI than their male counterparts. Oyejide and Osinusi^49^ earlier reported that under five male children experience ARI more than under five female children in the Idikan Community in Ibadan, Oyo state, Nigeria. Siziya et al.^35^ reported that under five male children are at a higher risk of diarrhoea than under five female children in Sudan. Jinadu et al.^47^ reported that male children are at a slightly higher risk of diarrhoea in rural areas of Akoko North, Ondo state, Nigeria. We found that male children are at a higher risk of diarrhoea and fever than ARI. The opposite is the case for female children. Children dwelling in urban areas are at a higher risk of ARI but lower risk of diarrhoea and fever compared to those in rural areas. The reverse holds for children in rural areas.

Children from the Northwest are at a higher risk of fever but a lower risk of diarrhoea than ARI. Children from the Southeast and Northwest are exposed to the same level of risk of ARI. Children from the North Central have a lower risk of ARI, while children from the Northeast, Southwest, and South-South have a higher risk of ARI than their counterparts in the Northwest. This contradicts^25^ whose descriptive results show that ARI is significantly higher among preschool children in the Northwestern zone of Nigeria than in the South-Southern zone of Nigeria. However, our results are partially consistent with^31^ who reported that under five children in the Northeastern and Northwestern regions of Nigeria are exposed to a higher risk of ARI because of higher exposure to dust levels and geographical locations of these areas along the Gulf of Guinea allowing for common sand storms^50,51^. Our results show that under five children in the South-South run the highest risk of ARI followed by their mates in the Southwest. Perhaps this could be mainly due to the sustained air pollution in those areas particularly in the South-South. Children in the Northeast have a higher risk of fever than ARI. Children from the North Central, Southeast, South-South, and Southwest have a lower risk of fever than ARI compared to children from the Northwest. Children from the North Central and Northeast are at a higher risk of diarrhoea. Children from the Southeast, South-South, and Southwest are at a lower risk of diarrhoea than ARI compared to children from the Northwest. As mentioned earlier, our results contradict^38^. We suspect that these variations could be attributed to changes in children’s immunization patterns nationwide (particularly, for fever and ARI, since the rotavirus vaccine for the prevention of the commonest cause of diarrhoea in children was only introduced into routine immunization in August 2022, see https://www.gavi.org/vaccineswork/dealing-diarrhoea-nigeria-introduces-rotavirus-vaccine-its-immunisation-plan, regional disparities in immune resilience or vulnerability, current climate conditions, economic downturns, inadequate urban planning, insufficient basic amenities like clean water and medical services, environmental degradation in oil-rich regions, displacement caused by insurgency in the North, and conflicts between Fulani herders and farmers in various parts of Nigeria.

Children whose mothers have primary or secondary education have a higher risk of ARI than children whose mothers have more than secondary education. Children whose mothers have primary or secondary education are at a lower risk of ARI compared to children whose mothers have no education. In addition to the list of ARI pathogens in Section 2, studies have shown that indoor air pollution caused by domestic cooking with biomass fuel or stove, both of which are linked to poverty, is a risk factor for ARI^52^. The recurrence of seasonal meningitis is due to ecological, economic, and socio-cultural factors^53^. Ecological factors include poorly designed built environments, crowded sleeping conditions, poorly ventilated rooms, dry weather, and dusty surroundings. Economic factors encompass poverty and neighborhood deprivation. Socio-cultural factors involve poor healthcare-seeking behaviors, social mixing patterns, inadequate vaccination, and vaccine hesitancy^54^. Similarly, the burning of biomass fuel and the emission of particulate matter (PM2.5) increases the risk of ARI in under five children^31,55–57^. Also,^58^ reported high emissions of PM2.5 in Nigeria from different anthropogenic sources like automobiles and fossil fuel combustion in heavily industrial and commercial locations as well as from the Sahara desert in the North. According to Eghomwanre & Oguntoke^59^ people in Benin City, Nigeria, whose kitchens are located indoors and cook with kerosene reported more childhood asthma (a respiratory disease) than those whose kitchens are located outdoors and cook with firewood and natural gas. In Uganda, a lower risk of under five ARI was reported for cooking with charcoal^46^. In Nigeria, rural dwellers are more inclined to cook with firewood or charcoal in an open space or under the canopy while the poor urban dwellers cook in their sometimes poorly ventilated kitchen within the confines of their apartment. Therefore, it could be argued that rural areas are largely characterized by cleaner air due to their greener nature compared to urban areas, except in a few places, like in the rural South-South, as a result of the environmental degradation occasioned by oil exploration and exploitation in that area. The health and environmental impact of the oil and gas drilling in the South-South of Nigeria (Niger Delta region) is well documented in the literature. Recent studies include^60–68^ and^69^. Again, it could be argued that uneducated mothers are more likely to be found in rural areas than educated mothers. Mothers with more than secondary education are less likely to reside in environmentally compromised parts of urban areas. We earlier found that ARI is more likely to affect children in urban areas than in rural areas so these two results resonate. Children whose mothers have no education are generally at a higher risk of diarrhoea and fever than ARI and this is similar to what was found in the Arba Minch district of Southern Ethiopia by Mohammed & Tamiru^70^ who found that children whose mothers have no education are at a higher risk of diarrhoea compared to children whose mothers have any form of education. Children of mothers who either have primary or secondary education are at a lower risk of diarrhoea and fever than ARI. Children whose mothers have more than secondary education are at a lower risk of diarrhoea but higher risk of fever than ARI compared to children whose mothers have no education.

Children from the richest homes (fifth wealth quintile) are at a higher risk of ARI but lower risk of diarrhoea and fever. Children whose families fall under the fifth wealth quintile, except the middle wealth quintile, are at a higher risk of diarrhoea than ARI compared to children coming from the richest families. Children whose families fall under the fifth wealth quintile except the second wealth quintile are at a higher risk of fever than ARI compared to children in the fifth wealth quintile. Abegunde et al.^71^ found that the odds of fever among under five children in Bauchi state increases with the higher socio-economic status of households. However, fever and ARI are equally likely to manifest among children from the second and fifth wealth quintiles. In the North Central, children from Benue and Niger states have a lower risk of fever while children from Kwara and Kogi states are at a higher risk of fever compared to children from the FCT. Children from Nasarawa and Plateau states are equally likely to experience fever compared to children from the FCT. Children from Kogi state are at a lower risk of diarrhoea than fever. Children from Benue, Kwara, Nasarawa, Niger and Plateau states are at a higher risk of diarrhoea than fever compared to children from the FCT.

In the Northeast, we found that children are exposed to the same level of risk of fever and diarrhoea in Adamawa state. Children in Taraba and Adamawa states share the same level of risk of fever. Children domiciled in Bauchi and Yobe states are at a lower risk of fever. Children domiciled in Gombe and Borno states are at a higher risk of fever compared to children domiciled in Adamawa state. Children from Bauchi, Taraba and Yobe states are at a higher risk of diarrhoea than fever. Children from Borno and Gombe states are at a lower risk of diarrhoea than fever compared to children from Adamawa state.

In the Northwest, we found that children from Zamfara state have a lower risk of diarrhoea and children from Zamfara and Kebbi states are exposed to an equivalent risk of fever. Children from Kastina, Kaduna, Sokoto, Jigawa and Kano states have a lower risk of fever than their counterparts in Zamfara state. Children from Jigawa, Kaduna, Kano, Katsina, Kebbi and Sokoto states have a higher risk of diarrhoea compared to their counterparts in Zamfara state.

In the Southeast, we found that children are at a higher risk of diarrhoea than fever in Abia state and the risk of fever among children in Abia and Enugu states are the same. Children are at a lower risk of fever in Anambara state. They are at a higher risk of fever in Ebonyi and Imo states compared to Abia state. Children are at a lower risk of diarrhoea compared to fever in Ebonyi and Imo states than in Abia state. Children in Anambara and Enugu states are exposed to a higher risk of diarrhoea than fever compared to children in Abia state.

In the South-South, we found that children have a lower risk of diarrhoea in Bayelsa state. Children in Edo and Bayelsa states are exposed to the same level of risk of fever. Children from Delta, Rivers and Cross Rivers states have a lower risk of fever. Children from Akwa Ibom state have a higher risk of fever than children from Bayelsa state. Children from Cross Rivers, Delta, Edo and Rivers states are at a higher risk of diarrhoea than fever. Children from Akwa Ibom state have a lower risk of diarrhoea than fever compared to children from Bayelsa state.

In the Southwest, children are exposed to equal risk of diarrhoea and fever in Lagos state. Children in Ondo and Lagos states are exposed to the same level of risk of fever. Children in Oyo state have a lower risk of fever than their mates in Lagos state. Children from Ekiti, Ondo and Ogun states are at a higher risk of fever than their mates in Lagos state. Children are exposed to an equal amount of risk of diarrhoea and fever in Lagos, Ekiti, Ogun, Ondo and Osun states. Children from Oyo state have a higher risk of diarrhoea than fever compared to their mates in Lagos state.

The disparity in children’s susceptibility to diarrhoea in Nigeria could be due to the disparity in mother’s education level and employment status as well as family income level across the states^22^, and the other reasons could be differences in hygiene and sanitation level of households^72^. Immunization prevents about 2 to 3 million of under five children deaths worldwide but in Nigeria, only about 25% of children between 12 to 23 months are fully immunized^73^. Therefore, the notable prevalence and spatial disparity in child morbidity in Nigeria could perhaps be due to variations in parents’ attitudes towards children vaccination or immunization (for only fever and ARI) in different geopolitical zones and states hinging on a wide array of factors such as parents hesitancy, delay or even refusal to vaccinate their children based on personal belief or conspiracy theory, safety concerns, inadequate information from healthcare providers, reliance on traditional remedies, cultural and religious beliefs, and viewing vaccination as a foreign concept^74,75^. In Oyo state (Southwest), many mothers of under five children failed to immunize their children due to long waiting times in queues at the immunization centers, financial implications in private clinics, and distance to the immunization centers^76^. Sometimes, the negative attitude of some healthcare workers could discourage mothers from following up on immunization programs^77^. In Nigeria, a positive relationship between mothers’ educational attainment and child immunization, as well as a relationship between religious affiliation and child immunization, have been found. Literate mothers are more likely to understand and remember health information and be aware of the availability and advantages of child immunization. Muslims are associated with lower odds of full immunization^73^. The poor utilization of immunization services among Muslim faithful may have a cultural undertone in addition to their circumspection on vaccinations^78^. Insufficient access to information and poor knowledge of mothers on routine immunization militates against the immunization uptake of under five children in Nigeria, for instance, in the rural Northwest the uptake and knowledge of immunization among caregivers are poor^79^ and specifically in Kaduna still in the Northwest, many mothers neither do not have nor have inadequate information on routine immunization with radio being the major source of information on child immunization^80^. By global standards, full vaccination among children remains arbitrarily low in Nigeria due to socioeconomic, socio-demographic, and religious factors for instance; children from wealthier families, living in southern regions, having Christian parents, belonging to Igbo or Yoruba ethnic origins, having mothers who attended five or more antenatal care appointments, giving birth in an institution, or having highly educated mothers are more likely to be fully vaccinated^81^. The issue of childhood immunization hesitance and non-compliance among young mothers between the ages of 17 and 23 years who had no post-secondary education and seldom attended immunization centers due to the subtle hostility and embarrassment they experienced during and after their pregnancy have also been reported in Ibadan in the Southwest^75^. In addition to poor immunization, other risk factors of under five morbidity, particularly ARIs were identified in Enugu state (Southeast). They included inadequate breastfeeding, attendance to daycare centers, large family size, poor educational attainment of the parents, smoking parents, urban dwelling, and the use of biofuels for cooking^82^. Many of these risk factors can be modified to abate infant mortality in Nigeria.

The findings herein can help the government and non-governmental bodies to design an effective intervention strategy in terms of aid distribution and allocation; training and retraining of healthcare workers to provide timely, unbiased, and accurate information to the parents; sensitization and awareness campaigns; support for women’s literacy and so on which are targeted at the ultimate reduction of child morbidity (ARI, diarrhoea, and fever) in Nigeria.

## Limitations

This paper cannot be complete without highlighting some of its potential limitations. First, we used secondary data, which constrained us to just a few variables to include in our analysis subject to data availability. Secondly, this paper relied only on the information from women in the household. So, there is a high tendency for bias due to under/over-reporting, and the level of bias may differ across various geopolitical zones and states. Finally, considering that we have used cross-sectional data, it is not possible for us to establish a cause-effect relationship.

## Data Availability

The data are freely available in the public domain, see “Data” section.

## Code availability

The code used can be obtained from the corresponding author.
